# Effects of high-dose vitamin D supplementation on bone mineral density in very low birth weight preterm infants

**DOI:** 10.3389/fendo.2025.1585898

**Published:** 2025-08-01

**Authors:** Myoung-Jin Yoo, Jeong Ho Seo, Inwook Lee, Hyuna Kim, Jinjoo Choi, Yunsoo Choe, Hyun Ju Lee, Seung Yang

**Affiliations:** ^1^ Department of Pediatrics, Hanyang University College of Medicine, Seoul, Republic of Korea; ^2^ Department of Pediatrics, Hanyang University Hospital, Seoul, Republic of Korea

**Keywords:** vitamin D, bone mineral density, VLBW (very low birth weight), DEXA, preterm

## Abstract

**Background:**

Vitamin D deficiency is common among very low birth weight (VLBW) preterm infants due to challenges in achieving adequate enteral nutrition and reduced transplacental transfer. Supplementation with 800 IU/day of vitamin D has been shown to safely and effectively increase serum 25-hydroxyvitamin D [25(OH)D] levels above 30 ng/mL when initiated at two weeks of age and continued until hospital discharge.

**Objective:**

This study aimed to evaluate whether daily supplementation with 800 IU of vitamin D significantly improves bone mineral density, as measured by dual-energy X-ray absorptiometry (DEXA), in VLBW infants at discharge, compared to supplementation with 400 IU/day.

**Methods:**

We retrospectively reviewed the medical records of 215 VLBW infants with birth weights under 1,500 g who were admitted to the neonatal intensive care unit at Hanyang University Seoul Hospital between January 2010 and December 2023. The infants were divided into two groups based on their daily vitamin D intake of either 400 IU or 800 IU, initiated on day 14 of life after trophic feeding was established. Bone mineral apparent density (BMAD) was assessed at discharge using whole-body DEXA (Hologic QDR-4500, infant mode).

**Results:**

Maternal age (32.01 ± 3.43 vs. 33.34 ± 4.39 years, *p* = 0.017) and birth weight (812.86 ± 141.19 vs. 883.76 ± 260.09 g, *p* = 0.010) were significantly higher in the 800 IU group, which also had a longer duration of total parenteral nutrition. After adjusting for birth weight and duration of parenteral nutrition, total body BMAD was significantly higher in the 800 IU group (*p* = 0.008). A general linear model further demonstrated that 800 IU supplementation was positively associated with femoral BMAD at discharge (β = 0.267, *p*= 0.001).

**Conclusion:**

Daily supplementation of 800 IU of vitamin D was associated with improved bone mineralization, as measured by DEXA, at discharge in VLBW infants.

## 1 Introduction

Vitamin D deficiency is highly prevalent among very low birth weight (VLBW) preterm infants due to challenges in achieving adequate enteral nutrition, reduced transplacental transfer, limited ultraviolet B exposure, and low fat mass for vitamin D storage (1).

Adequate vitamin D intake is essential for the development of bone mineral density (BMD) and overall growth in these infants (2). Low BMD in preterm infants is associated with an increased risk of skeletal complications, such as rickets and osteopenia of prematurity (OOP). These conditions can be evaluated using clinical, biochemical, and radiological assessments. Common biochemical markers include serum calcium, phosphate, alkaline phosphatase (ALP), and 25-hydroxyvitamin D (25(OH)D). In addition, radiological methods such as wrist X-rays and dual-energy X-ray absorptiometry (DEXA) are critical for evaluating bone health (2). While DEXA is a precise tool for predicting metabolic bone disease, its utility is enhanced when combined with clinical and biochemical markers, given its limitations (3).

Vitamin D plays a fundamental role in bone health by enhancing calcium and phosphate absorption through the regulation of sodium-phosphate co-transporters (NaPi-IIb) and transient receptor potential vanilloid 6 (TRPV6) in enterocytes. Activated vitamin D binds to the vitamin D receptor (VDR) in osteoblasts, stimulating the expression of RANKL (Receptor Activator of Nuclear Factor Kappa-β Ligand), which promotes bone remodeling and osteoclast differentiation. Additionally, vitamin D upregulates ALP and osteocalcin, both of which are essential for osteoblast-mediated bone mineralization (4). This molecular mechanism suggests that higher vitamin D intake in preterm infants may reduce the risk of OOP and improve bone mineralization.

The optimal dosage and timing of vitamin D supplementation in preterm infants remain subjects of ongoing investigation. European guidelines recommend 800–1,000 IU/day, while U.S. guidelines commonly suggest 400 IU/day (5, 6). However, recent studies have questioned the adequacy of the 400 IU dose, suggesting that it may not be sufficient to support optimal bone health in high-risk preterm populations. Our previous research demonstrated that 800 IU of vitamin D safely and effectively raised 25(OH)D levels above 30 ng/mL in VLBW infants from two weeks of age until discharge (7).

This study aims to evaluate whether a daily vitamin D dose of 800 IU significantly improves BMD, as measured by DEXA, in VLBW infants at discharge compared to a 400 IU dose.

## 2 Methods

### 2.1 Study design and participants

This retrospective cohort study included 215 very low birth weight infants (VLBWIs; birth weight <1,500 g) admitted to the NICU at Hanyang University Seoul Hospital between January 2011 and December 2022. Following the implementation of a high-dose vitamin D protocol in September 2017, infants were categorized into two groups: 400 IU/day (n = 70; Period 1: January 2011–October 2015) and 800 IU/day (n = 145; Period 2: November 2015–December 2022).

Liquid cholecalciferol (vitamin D_3_; Sunny D^®^, GMP Laboratories, USA) was administered via a nasogastric or orogastric tube starting on day 14 of life if enteral feeding was tolerated, and continued until 36 weeks postmenstrual age (PMA) (8). Medical records for Period 1 were retrospectively reviewed with an IRB waiver of consent, while informed consent was obtained for infants in Period 2 (IRB No. 08028019).

### 2.2 Nutritional protocol and vitamin D supplementation

Once infants achieved 100 mL/kg/day of enteral feeding, they received either fortified human milk (containing 82 mg calcium, 45 mg phosphorus, and 100 IU vitamin D per 100 mL) or mineral-fortified preterm formula (115 mg calcium, 62 mg phosphorus, and 66 IU vitamin D per 100 mL). Total daily vitamin D intake, including supplementation, was limited to 900 IU/day. Supplementation continued until 36 weeks PMA and was discontinued if serum 25(OH)D exceeded 80 ng/mL. In the 800 IU/day group, 25(OH)D levels were measured at birth (cord blood), and at 32 and 36 weeks PMA.

### 2.3 NICU fluid and feeding protocol

Initial fluid was administered at 60–80 mL/kg/day and advanced to ≥120 mL/kg/day within 7 days. Enteral feeding began on days 1–2 at 10 mL/kg/day and was advanced based on tolerance. Glucose was started at 6–8 g/kg/day and increased up to 17–18 g/kg/day. Amino acids and lipids were initiated at 1.5–2 g/kg/day and progressed to 3–3.5 g/kg/day. The non-protein calorie-to-nitrogen ratio was maintained between 150:1 and 200:1. These TPN and feeding protocols remained consistent throughout the study period, supporting comparability between groups and minimizing potential temporal bias (9).

### 2.4 Other bone health interventions

Throughout the study period, standard neurodevelopmental physical therapy (NPT) practices were consistently applied as part of routine NICU care. These included daily gentle passive range-of-motion (ROM) exercises, facilitated positioning, and developmental handling techniques to promote musculoskeletal development and prevent disuse-related bone loss. Such mobilization strategies have been shown to positively affect bone mineralization and somatic growth in VLBW infants, as supported by previous randomized trials. Importantly, there were no major changes in the mobilization or rehabilitation protocols between the two study periods, minimizing the risk of confounding due to evolving physical therapy practices (10).

### 2.5 Data collection and outcome measures

Maternal and neonatal baseline variables (e.g., maternal age, hypertension, gestational diabetes, oligohydramnios, birth weight/height, Apgar scores, mode of delivery) and postnatal morbidities (e.g., respiratory distress syndrome [RDS], patent ductus arteriosus [PDA], intraventricular hemorrhage [IVH], retinopathy of prematurity [ROP], bronchopulmonary dysplasia [BPD], postnatal corticosteroid use) were collected. Biochemical parameters (serum calcium, phosphorus, ALP) were monitored weekly.

### 2.6 Definitions of prematurity-related conditions

Assessed neonatal morbidities included respiratory distress syndrome (RDS), patent ductus arteriosus (PDA), retinopathy of prematurity (ROP, ≥ stage II), bronchopulmonary dysplasia (BPD, ≥ moderate), postnatal corticosteroid use, intraventricular hemorrhage (IVH, ≥ grade 2), duration of total parenteral nutrition (TPN), and length of hospitalization.

ROP ≥ stage II was defined according to the International Classification of ROP (10); BPD was defined as oxygen dependency ≥21% at 36 weeks PMA (11); and IVH ≥ grade 2 was diagnosed based on Papile’s criteria (11).

### 2.7 Bone mineralization assessment

At discharge, bone mineralization was assessed using DEXA (Hologic QDR-4500, whole-body infant mode) (2). Bone area (BA), bone mineral content (BMC), and bone mineral density (BMD) were measured for the whole body, spine, and femurs. Bone mineral apparent density (BMAD) was calculated by dividing BMD by the square root of BA.

Infants were categorized into BMAD groups as follows: good (>75th percentile, >0.008 g/cm³), fair (25th–75th percentile), and poor (<25th percentile, <0.006 g/cm³). These cutoff values were based on the interquartile distribution of our institutional cohort of VLBW infants who underwent DEXA scans during the study period, given the absence of standardized BMAD reference data for preterm populations. This percentile-based classification approach is consistent with that used by Lee et al. in a previous study of ELBW infants, supporting the validity of using internal cohort distributions for BMAD interpretation in the absence of external normative standards (3) (12–14).

DEXA scans were performed using standardized prone positioning with gentle limb extension and swaddling to minimize motion artifacts. Repeat scans were obtained if any movement was detected during acquisition. Despite these precautions, minor asymmetries in femur alignment or leg rotation may have introduced small degrees of site-specific variability, particularly in femoral measurements, as previously noted in neonatal DEXA guidelines (3, 13).

### 2.8 Statistical analysis

Clinical characteristics and bone parameters were compared using Student’s t-test or the χ² test. Group differences in DEXA outcomes were analyzed using a general linear model adjusted for birth weight and TPN days. Additionally, estimated marginal means (EMMs) were derived from a weighted linear mixed model (LMM) applied to IPTW-adjusted data to simultaneously account for repeated measurements across body regions and baseline covariate imbalance.

To minimize baseline differences between groups, inverse probability of treatment weighting (IPTW) using propensity scores was performed based on maternal age, birth weight, 5-minute Apgar score, postnatal corticosteroid use, and duration of TPN. For comparisons among poor BMAD, fair BMAD, and good BMAD, one-way ANOVA followed by least significant difference (LSD) *post hoc* testing was conducted to identify pairwise differences.

## 3 Results

A total of 215 very low birth weight infants (VLBWIs) were included and divided into two groups based on early vitamin D supplementation: 400 IU/day (n=70) and 800 IU/day (n=145). As shown in **Table 1**, before inverse probability of treatment weighting (IPTW), several baseline characteristics differed significantly between groups. The 800 IU/day group had significantly higher maternal age, birth weight, birth height, cesarean section rate, Apgar scores at 1 and 5 minutes, and total TPN days, while postnatal corticosteroid use and necrotizing enterocolitis (NEC) incidence were more frequent in the 400 IU/day group.

**Table 1:** Clinical characteristics of the infants.

	Unweighted study population			Weighted study population		
	Vitamin D (400 IU/day) (N=70)	Vitamin D (800 IU/day) (N=145)	*p*	Vitamin D (400 IU/day)	Vitamin D (800 IU/day)	*p*
Maternal characteristics						
Maternal age, year	32.01 ± 3.43	33.33 ± 4.41	0.018	33.25 ± 0.83	33.32 ± 0.41	0.935
Hypertension	9 (12.9)	10 (6.9)	0.149	24.5 (13.8)	22.22 (9.7)	0.570
Gestational diabetes	4 (5.7)	7 (4.8)	0.782	6.84 (3.8)	8.39 (3.7)	0.947
Oligohydramnios	4 (5.7)	8 (5.5)	0.953	5.27 (3.0)	10.76 (4.7)	0.465
Infant characteristics						
Gestational age, weeks	25.99 ± 2.12	26.56 ± 3.11	0.115	26.10 ± 0.40	26.78 ± 0.42	0.246
Birth weight, g	812.86 ± 141.19	883.65 ± 260.99	0.011	839.26 ± 22.04	880.88 ± 35.06	0.316
Birth height, cm	32.99 ± 2.60	34.20 ± 4.66	0.015	33.44 ± 0.38	34.32 ± 0.61	0.224
Birth head circumference, cm	23.94 ± 2.62	24.45 ± 3.00	0.204	24.33 ± 0.36	24.79 ± 0.44	0.418
Cesarean section	43 (61.4)	114 (78.6)	0.008	99.83 (56.1)	181 (79.5)	0.013
Male sex	26 (37.1)	74 (51.0)	0.056	86.62 (48.7)	122.60 (53.6)	0.632
Apgar score at 1 min	1.63 ± 0.95	2.65 ± 1.66	<0.001	1.95 ± 0.15	2.36 ± 0.17	0.065
Apgar score at 5 min	3.56 ± 1.38	5.47 ± 1.65	<0.001	4.43 ± 0.31	4.85 ± 0.27	0.303
RDS	68 (97.1)	137 (94.5)	0.385	171.16 (96.2)	210.71 (92.2)	0.427
PDA	53 (75.7)	121 (83.4)	0.176	122.43 (68.8)	176.05 (77.0)	0.418
NEC	9 (12.9)	25 (17.2)	0.282	36.4 (20.5)	33.6 (14.7)	0.257
IVH ≥stage II	15 (21.4)	19 (13.1)	0.117	30.53 (17.2)	32.54 (14.2)	0.684
ROP ≥stage II	23 (32.9)	54 (37.5)	0.507	60.47 (34.0)	76.79 (33.6)	0.966
BPD ≥moderate	31 (44.3)	56 (38.6)	0.428	62.31 (35.0)	79.91 (35.0)	0.993
Postnatal corticosteroid	48 (68.6)	47 (32.4)	<0.001	35.04 (19.7)	57.04 (24.9)	0.490
Hospital days	95.77 ± 26.63	99.05 ± 41.64	0.486	92.29 ± 3.96	102.37 ± 6.95	0.209
TPN days	36.60 ± 15.37	52.31 ± 29.53	<0.001	39.19 ± 3.89	47.84 ± 3.13	0.085

Data are expressed as means ± standard deviations or n (%). BPD, bronchopulmonary dysplasia; TPN, total parenteral nutrition; RDS, respiratory distress syndrome; PDA, patent ductus arteriosus; IVH, intraventricular hemorrhage; ROP, retinopathy of prematurity; NEC, necrotizing enterocolitis.

IPTW was applied using propensity scores estimated from maternal age, birth weight, Apgar score at 5 minutes, postnatal corticosteroid use, and TPN days. After weighting, all standardized mean differences (SMDs) were below 0.2, except for TPN days (SMD = 0.227), indicating acceptable covariate balance between groups. IPTW-adjusted *p*-values are presented in the right columns of **Tables 1**, **2**. Linear mixed model estimates using IPTW-weighted data (**Table 3**) were presented as a complementary approach to support the consistency of the findings.

**Table 2:** Comparison of bone mineral density provided with 400 IU/day and 800 IU/day vitamin D supplementation.

	Vitamin D (400 IU/day) (N=70)	Vitamin D (800 IU/day) (N=145)	*p*	*Weighted p* ^a^ *(IPTW)*
BA, cm^2^	312.87 ± 78.93	295.43 ± 54.78	0.061	0.472
BMC, mg	38.79 ± 26.95	37.78 ± 15.75	0.730	0.604
BMD, mg/cm^2^	0.12 ± 0.03	0.13 ± 0.03	0.117	**0.013**
BMAD, mg/cm^3,*^	0.67 ± 0.13	0.73 ± 0.17	0.005	**<0.001**
Spine				
BA	33.11 ± 9.10	31.24 ± 9.60	0.175	0.174
BMC	3.51 ± 1.39	3.53 ± 1.62	0.904	0.678
BMD	0.10 ± 0.02	0.11 ± 0.03	0.049	**0.015**
BMAD^*^	1.86 ± 0.45	2.01 ± 0.44	0.024	**0.002**
Right femur				
BA	10.00 ± 2.47	9.99 ± 2.52	0.979	0.816
BMC	1.01 ± 0.28	1.10 ± 0.38	0.078	**0.017**
BMD	0.10 ± 0.02	0.12 ± 0.07	0.116	**0.006**
BMAD^*^	3.35 ± 0.84	3.57 ± 0.91	0.086	0.104
Left femur				
BA	10.99 ± 4.02	10.16 ± 2.03	0.107	0.361
BMC	1.15 ± 0.52	1.19 ± 0.37	0.596	0.879
BMD	0.10 ± 0.02	0.12 ± 0.03	0.001	**0.006**
BMAD^*^	3.25 ± 0.78	3.69 ± 0.84	<0.001	**0.027**

Data are expressed as means ± standard deviations or n (%). BA, bone area; BMC, bone mineral content; BMD, bone mineral density; BMAD, bone mineral apparent density; IPTW, inverse probability of treatment weighting; TPN, total parental nutrition.

a Weighted p-values were obtained using IPTW based on propensity scores estimated from maternal age, birth weight, Apgar score at 5 minutes, dexamethasone use, and TPN days.

^*^ BMAD is expressed as means ± standard deviations (10^−2^).

Bold indicates statistical significance at p < 0.05.

**Table 3:** Comparison of bone mineral density provided with 400 IU/day and 800 IU/day vitamin D supplementation using estimated marginal means derived from a weighted linear mixed effects model applied to IPTW-adjusted data.

Vitamin D (400 IU/day) vs Vitamin D (800 IU/day)				
Location	Estimate	Std. Error	t	p*
BMAD	0.001	0.001	0.610	0.542
BMADs	0.001	0.001	1.447	0.148
BMADrf	0.002	0.001	2.566	**0.010**
BMADlf	0.004	0.001	4.640	**<0.001**

BMAD, bone mineral apparent density; BMADs, at spine; BMADrf, at right femur; BMADlf, at left femur.

*adjusted for gestational age, birth heights, postnatal corticosteroid use, head circumference at densitometry, and inverse probability of treatment weighting (IPTW).

Bold indicates statistical significance at p < 0.05.

Although a statistically significant improvement was observed in the left femur BMAD, this may partly reflect positioning or measurement variability in very small preterm infants. However, consistent patterns were observed across other skeletal sites, supporting a general beneficial effect of higher-dose vitamin D supplementation. IPTW-adjusted analyses further strengthened the robustness of these findings.

After IPTW, clinical differences in baseline characteristics were no longer statistically significant, except for TPN days (*p*=0.085), which remained slightly imbalanced. In bone densitometry analyses, the 800 IU/day group showed significantly higher whole-body BMAD compared to the 400 IU/day group even after IPTW adjustment (weighted *p* < 0.001). Similar but less significant patterns were observed in the spine and femoral regions: spine BMAD (weighted *p* = 0.002), right femur BMAD (*p* = 0.104), and left femur BMAD (*p* = 0.027), indicating a consistent positive effect of higher-dose vitamin D supplementation on skeletal mineralization across multiple sites.

To assess the biochemical safety of the high-dose regimen, serum 25(OH)D levels were monitored in the 800 IU/day group. The mean cord blood level was 29.7 ng/mL, and the mean level at discharge was 43.8 ng/mL. Three infants exceeded 80 ng/mL prior to discharge, with cord blood levels of 24.2, 27.4, and 35.2 ng/mL, and discharge levels of 91.6, 99.6, and 84.0 ng/mL, respectively. In all cases, vitamin D supplementation was promptly discontinued according to protocol, and no clinical signs of toxicity were observed, supporting the safety of the 800 IU/day regimen in this population. Detailed individual data are provided in **Supplementary Table X**.

Clinical characteristics according to BMAD classification are summarized in **Table 4**. Infants with good BMAD had a significantly higher gestational age (27.63 ± 3.81 weeks) compared to those with poor (25.57 ± 1.93 weeks) or fair BMAD (26.22 ± 2.58 weeks, *p* = 0.002). Birth height also showed significant differences among groups (good: 35.05 ± 4.70 cm, poor: 32.76 ± 3.03 cm, fair: 33.73 ± 4.18 cm, *p* = 0.037). Postnatal corticosteroid use was significantly lower in the good BMAD group (27.9%) than in the poor (59.5%) and fair (44.6%) groups (*p* = 0.013). The good BMAD group also showed a higher rate of vitamin D 800 IU supplementation (81.4% vs. 47.6% in poor and 69.2% in fair groups, *p* = 0.003). In laboratory findings, lowest serum calcium differed significantly between groups (good: 7.86 ± 1.28 mg/dL, poor: 7.06 ± 1.13 mg/dL, fair: 7.76 ± 1.19 mg/dL, *p* = 0.002). In bone densitometry results, good BMAD infants had significantly higher BMC (56.42 ± 34.57 g), BMD (16.80 ± 4.55 × 10^−2^), BMCs (4.74 ± 2.02 g), and BMDs (12.87 ± 5.27 ×10^−2^) compared to the other groups (all *p* < 0.001). These results indicate that higher vitamin D supplementation and greater gestational maturity may contribute to improved bone mineralization in very low birth weight infants.

**Table 4:** Clinical characteristics based on bone mineral apparent density.

	Poor BMAD^a^ (N=42)	Fair BMAD^b^ (N=130)	Good BMAD^c^ (N=43)	*p*	*LSD*
Maternal characteristics					
Maternal age, year	32.60 ± 3.66	32.85 ± 4.34	33.35 ± 4.07	0.691	
Hypertension	3 (7.1)	10 (7.7)	6 (14.0)	0.415	
Gestational diabetes	2 (4.8)	7 (5.4)	2 (4.7)	0.976	
Oligohydramnios	2 (4.8)	7 (5.4)	3 (7.0)	0.895	
Infant characteristics					
Gestational age, weeks	25.57 ± 1.93	26.22 ± 2.58	27.63 ± 3.81	0.002	a<c
Birth weight, g	798.02 ± 155.49	861.05 ± 21.802	920.35 ± 37.80	0.050	
Birth height, cm	32.76 ± 3.03	33.73 ± 4.18	35.05 ± 4.70	0.037	a<c
Birth head circumference, cm	23.71 ± 2.40	24.19 ± 2.87	25.12 ± 3.17	0.065	
Cesarean section	27 (64.3)	99 (76.2)	31 (72.1)	0.318	
Male sex	17 (40.5)	62 (47.7)	21 (48.8)	0.677	
Apgar score at 1 min	2.12 ± 1.42	2.22 ± 1.45	2.81 ± 1.83	0.056	
Apgar score at 5 min	4.55 ± 1.66	4.81 ± 1.81	5.26 ± 1.92	0.181	
RDS	38 (90.5)	127 (97.7)	40 (93.0)	0.112	
PDA	32 (76.2)	103 (79.2)	39 (90.7)	0.173	
ROP ≥stage II	9 (21.4)	53 (41.1)	15 (34.9)	0.069	
BPD ≥moderate	17 (40.5)	48 (36.9)	22 (51.2)	0.257	
Postnatal corticosteroid	25 (59.5)	58 (44.6)	12 (27.9)	0.013	a>c
TPN days	45.43 ± 20.78	48.42 ± 28.82	45.21 ± 25.88	0.709	
Lowest serum P, mg/dL	2.98 ± 130	3.23 ± 1.55	3.79 ± 1.13	0.025	a, b<c
Lowest serum Ca, mg/dL	7.06 ± 1.13	7.76 ± 1.19	7.86 ± 1.28	0.002	a<b, c
Peak ALP	690.18 ± 274.48	689.91 ± 339.00	589.63 ± 211.00	0.159	
Infant characteristics at densitometry					
Weight, g	2303.81 ± 428.62	2321.23 ± 474.59	2370.47 ± 630.33	0.807	
Height, cm	43.86 ± 3.21	43.28 ± 3.15	43.73 ± 5.10	0.588	
Head circumference, cm	30.28 ± 3.60	30.31 ± 4.25	32.54 ± 4.81	0.010	a, b<c
P	5.61 ± 1.07	5.84 ± 0.86	5.77 ± 0.95	0.349	
Ca	9.72 ± 0.45	9.80 ± 0.82	9.85 ± 0.81	0.719	
ALP	369.95 ± 139.31	375.18 ± 180.78	353.14 ± 149.98	0.756	
Bone mineral density					
BMC	31.04 ± 8.05	34.33 ± 10.66	56.42 ± 34.57	<0.001	a, b<c
BMD^*^	9.90 ± 1.48	11.65 ± 1.42	16.80 ± 4.55	<0.001	a<b<c
BMCs	3.17 ± 1.11	3.24 ± 1.25	4.74 ± 2.05	<0.001	a, b<c
BMDs^*^	9.55 ± 1.99	10.40 ± 1.90	12.87 ± 2.57	<0.001	a<b<c
**Vitamin D 800 IU**	20 (47.6)	90 (69.2)	35 (81.4)	0.003	a>b, c

Data are expressed as means ± standard deviations or n (%).

BMAD, bone mineral apparent density; BMD, bone mineral density; BMDs, at spine; BMC, bone mineral content; BMCs, at spine; BPD, bronchopulmonary dysplasia; TPN, total parenteral nutrition; Ca, calcium; P, phosphorus; ALP, alkaline phosphatase; LSD, limited state design.

Group comparisons were conducted using one-way ANOVA with LSD *post hoc* analysis.

Superscript letters (a, b, c) indicate significant *post hoc* differences (*p* < 0.05).

^*^ BMD is expressed as means ± standard deviations (10^−2^)

## 4 Discussion

Osteopenia of prematurity (OOP) remains a significant concern among very low birth weight (VLBW) infants, who are at high risk for impaired bone mineralization due to multiple factors, including inadequate mineral intake. Extensive research has emphasized the importance of vitamin D supplementation in preventing metabolic bone disease, including OOP, in this vulnerable population.

Initially, our standard regimen involved daily supplementation with 400 IU of vitamin D, a dose widely recommended for preterm infants at the time. However, a substantial proportion of infants remained vitamin D deficient despite this supplementation. Recognizing this gap, our previous research provided compelling evidence supporting the safety and efficacy of 800 IU/day of vitamin D, demonstrating marked improvements in both vitamin D status and bone mineralization (7).

In the present study, 52 preterm infants (mean gestational age: 27.1 weeks) received 800 IU/day of vitamin D starting at two weeks of age. Prior to supplementation, 41% of these infants were vitamin D deficient, and an additional 49% had insufficient levels. Following supplementation, no infants remained deficient, and 72% reached sufficient vitamin D levels by 36 weeks postmenstrual age (PMA). Notably, the higher dose was associated with a significantly lower incidence of OOP, underscoring the clinical importance of optimizing vitamin D intake in preterm infants.

Among the skeletal sites evaluated, the left femur showed the most pronounced improvement in BMAD with high-dose vitamin D supplementation. While this may partially reflect technical variability, such as subtle differences in positioning or leg rotation during scanning, similar trends were observed in the spine and right femur, supporting a generalized skeletal effect. Given that DEXA measurements of the femur are more susceptible to alignment artifacts than central sites like the spine, and that preterm infants often have asymmetric posture or low muscle tone, such variability is not unexpected. The ISCD pediatric guidelines acknowledge that small differences in limb position can affect regional DEXA measurements in this population (13). Importantly, bilateral femoral improvements were seen, with greater magnitude on the left, suggesting an exaggerated expression of an overall systemic effect rather than a clinically meaningful unilateral difference. Further research using bilateral imaging and adjunctive modalities such as quantitative ultrasound (QUS) may help validate this interpretation.

In the 400 IU/day supplementation group, serum 25(OH)D levels were not available due to the retrospective nature of the study and institutional practices at the time, which were aligned with ESPEN guidelines (15). These guidelines consider 400 IU/day to be a standard dose that does not require routine vitamin D monitoring in preterm infants. Instead, bone health was routinely assessed using wrist radiographs, discharge DEXA scans, and serial biochemical markers such as serum calcium, phosphorus, and alkaline phosphatase (ALP), which were regularly monitored throughout hospitalization.

### 4.1 Clinical evidence supporting higher vitamin D supplementation

A randomized controlled trial by Ann Anderson-Berry et al. demonstrated that preterm infants (<32 weeks gestational age) receiving 800 IU/day of vitamin D had significantly greater improvements in serum 25(OH)D levels compared to those receiving 400 IU/day (5). Additionally, the 800 IU/day group showed enhanced bone mineral density (BMD) by DEXA and better linear growth.

Vitamin D supplementation has also been associated with improved physical growth parameters (weight, length, BMI, and head circumference) and neurodevelopmental outcomes in NICU infants. Furthermore, studies have reported no increased risk of serious morbidities such as BPD or NEC with vitamin D supplementation (16). In our study, larger head circumference was significantly associated with higher BMD (*p*=0.01), supporting the role of vitamin D in skeletal and overall growth. Future long-term follow-up studies on growth and neurodevelopmental outcomes based on vitamin D supplementation are warranted.

Interestingly, the 800 IU/day group had a significantly longer duration of total parenteral nutrition (TPN) (52.31 ± 29.53 days vs. 36.60 ± 15.37 days, *p* < 0.001), possibly reflecting greater illness severity or delayed enteral feeding. Despite prolonged TPN, this group showed better bone mineralization outcomes, suggesting that high-dose vitamin D may confer protective effects even under suboptimal nutritional conditions. While residual confounding cannot be completely excluded, this finding highlights the robustness of the clinical benefit associated with 800 IU/day supplementation.

### 4.2 Safety considerations of 800 IU/day vitamin D supplementation

Despite growing evidence, some concerns remain regarding the safety of 800 IU/day supplementation in neonates. However, several studies have confirmed its safety. For example, Natalia Aristizabal et al. reported no evidence of vitamin D or calcium toxicity in extremely preterm infants (<28 weeks) receiving 800 IU/day (17). Similarly, Dominika et al. found no serious neonatal morbidities associated with high-dose vitamin D, although they noted a possible reduction in serum parathyroid hormone levels (18). Hein G et al. raised concerns about hypercalciuria and nephrocalcinosis with excessive vitamin D intake (19). Given these concerns, routine monitoring of serum calcium and 25(OH)D levels is essential in VLBW infants receiving high-dose supplementation to ensure safety and prevent complications.

### 4.3 Challenges in assessing bone health in preterm infants

Evaluating bone mineral density (BMD) and mineralization in preterm infants remains challenging, as there is no universally accepted gold standard. Current assessment methods include biochemical markers, radiologic imaging, and clinical evaluation. Although serum alkaline phosphatase (ALP) levels are commonly used to assess bone turnover in preterm infants, their diagnostic utility for OOP is limited.

Our previous study examined BMD and BMAD in extremely low birth weight infants at discharge. Among 70 infants included, with an average gestational age of 25.9 weeks and a birth weight of approximately 813 g, the poor BMD group had significantly lower BMD values in the lumbar spine compared to the other groups. Factors associated with low BMD included BPD, prolonged TPN, and elevated ALP levels (3).

### 4.4 DEXA as a bone health assessment tool in preterm infants

Williams et al. demonstrated the utility of dual-energy X-ray absorptiometry (DEXA) in accurately quantifying bone mineral content (BMC) and BMD in preterm infants (20). Similarly, Rigo et al. showed that preterm infants have significantly lower BMC and BMD values compared to term infants, highlighting their increased risk of osteopenia and fractures (21). These findings reinforce the importance of early detection and timely intervention.

### 4.5 Future directions: advancing bone health assessment

DEXA is currently the most validated method for assessing bone mineral density in preterm infants, especially in research settings. However, its limitations include the need for infant transport, exposure to radiation, and the lack of standardized reference values for VLBW infants (22). Further research is needed to validate QUS and establish reference standards that could enable safer and more accessible bone health monitoring in this population.

## 5 Conclusion

Our findings indicate that daily supplementation with 800 IU of vitamin D, initiated at 14 days of life, significantly enhances bone mineralization in very low birth weight (VLBW) preterm infants compared to the standard 400 IU dose. Bone mineral density (BMD), assessed using dual-energy X-ray absorptiometry (DEXA), was significantly higher in the 800 IU group at discharge, supporting the potential benefits of a higher vitamin D intake in this high-risk population.

## 6 Limitation

This study has several limitations. First, it was a single-center study conducted in a specific geographic region, which may limit the generalizability of the findings. Ethnicity, genetic factors, and maternal conditions such as gestational diabetes mellitus can affect neonatal vitamin D status (23). Given that vitamin D deficiency is more prevalent in Asian and Middle Eastern populations than in Western countries, further multicenter and international studies are needed to validate these results in more diverse populations.

Second, BMAD measurements were conducted only at the time of discharge. We did not assess BMAD before the initiation of vitamin D supplementation because the infants were extremely vulnerable, with birth weights under 1500 g and typically less than 2 weeks of age at the start of supplementation. These preterm infants could not be removed from their incubators or safely transported outside the NICU during this early period. Therefore, performing pre-supplementation BMAD scans was not feasible.

Additionally, serum 25(OH)D levels were not collected in the 400 IU/day group, as retrospective data collection reflected the institutional standard of care at the time. In accordance with ESPEN recommendations, vitamin D status was not routinely monitored at this dosage level (15). Bone health assessments were conducted through standard clinical practice using biochemical markers (calcium, phosphorus, ALP) and radiologic tools, including wrist X-rays and discharge DEXA scans.

Although DEXA is widely used to assess bone mineral density in preterm infants, it has inherent limitations. While DEXA involves minimal radiation exposure and is considered safe even in neonates, especially with modern devices such as the Hologic QDR-4500, reported effective doses are less than 1 μSv, which is lower than natural background radiation levels (24). However, the lack of standardized reference values for VLBW infants remains a critical challenge.

This study highlights the need for larger-scale randomized controlled trials and longitudinal studies to establish standardized protocols and reference values for bone health assessment in VLBW infants. A more comprehensive and clinically practical evaluation strategy is required to facilitate early detection of osteopenia, enabling timely intervention and improved long-term outcomes for this vulnerable population.

This study has certain limitations. The observed differences in Apgar scores likely reflect physiological immaturity rather than perinatal compromise. Recent neonatal resuscitation practices have placed greater emphasis on immediate stabilization instead of Apgar-based assessment, which may have affected these scores (25). The direct application of monitoring devices in the NICU also influenced initial evaluations.

Throughout the study, the TPN protocol and parenteral nutrition composition remained consistent, with no major changes in feeding advancement strategies, mineral supplementation, or other bone health interventions. The only notable change during Period 2 (2015–2022) was the implementation of routine vitamin D level monitoring and the increased supplementation dose. While improvements in overall neonatal care over time may have contributed to better outcomes, the observed enhancements in bone mineralization were temporally aligned with the change in vitamin D practices, supporting a specific effect beyond general advances in NICU care.

Future research should address these limitations through well-designed, prospective randomized controlled trials with adequate sample size calculations. Serial monitoring of vitamin D status, including repeated 25(OH)D measurements, should be incorporated to better evaluate biochemical efficacy. Detailed reporting of gastrointestinal morbidities and corticosteroid exposure is also necessary to reduce potential confounding. Furthermore, the development of standardized reference values for DEXA interpretation in preterm populations, along with the validation of alternative methods such as quantitative ultrasound, is essential. Long-term follow-up beyond hospital discharge will be critical in elucidating the sustained effects of early vitamin D supplementation on bone health and growth in VLBW infants.

## Data Availability

The original contributions presented in the study are included in the article/**Supplementary Material**. Further inquiries can be directed to the corresponding author.
