# Supplementary material for: Effects of high-dose vitamin D supplementation on bone mineral density in very low birth weight preterm infants
Source: Front Endocrinol (Lausanne). 2025 Aug 1;16:1585898. doi: 10.3389/fendo.2025.1585898 (PMC12355404; doi:10.3389/fendo.2025.1585898)
Supplement: Supplementary file 1 [file Table1.docx]

**Supplement Table X**. Serum 25(OH)D concentrations in three infants exceeding 80 ng/mL with high-dose vitamin D supplementation

| **Patient** | **Cord 25(OH)D (ng/mL)** | **Discharge 25(OH)D**  **(ng/mL)** | **Action taken** | **Clinical Toxicity** |
| --- | --- | --- | --- | --- |
| 1 | 24.2 | 91.6 | Vitamin D stopped | None |
| 2 | 27.4 | 99.6 | Vitamin D stopped | None |
| 3 | 35.2 | 84.0 | Vitamin D stopped | None |
